# Therapeutic Effects of Treatment with Anti-TLR2 and Anti-TLR4 Monoclonal Antibodies in Polymicrobial Sepsis

**DOI:** 10.1371/journal.pone.0132336

**Published:** 2015-07-06

**Authors:** Cristiano Xavier Lima, Danielle Gloria Souza, Flavio Almeida Amaral, Caio Tavares Fagundes, Irla Paula Stopa Rodrigues, Jose Carlos Alves-Filho, Marie Kosco-Vilbois, Walter Ferlin, Limin Shang, Greg Elson, Mauro Martins Teixeira

**Affiliations:** 1 Faculdade de Medicina, Universidade Federal de Minas Gerais, Av. Prof. Alfredo Balena 190, sala 203, 30130–100, Belo Horizonte, MG, Brasil; 2 Instituto de Ciências Biologicas, Universidade Federal de Minas Gerais, Av. Presidente Antônio Carlos 6627, 31270–901, Belo Horizonte, MG, Brasil; 3 Faculdade de Medicina de Ribeirão Preto, USP Avenida dos Bandeirantes, 3.900, Monte Alegre, 14049–900, Ribeirão Preto, SP, Brasil; 4 NovImmune SA, 14 Chemin des Aulx, 1228 Plan-Les-Ouates, Geneva, CH1228, Switzerland; French National Centre for Scientific Research, FRANCE

## Abstract

**Introduction:**

Toll-like receptors (TLRs) play an important role in the recognition of microbial products and in host defense against infection. However, the massive release of inflammatory mediators into the bloodstream following TLR activation following sepsis is thought to contribute to disease pathogenesis.

**Methods:**

Here, we evaluated the effects of preventive or therapeutic administration of monoclonal antibodies (mAbs) targeting either TLR2 or TLR4 in a model of severe polymicrobial sepsis induced by cecal ligation and puncture in mice.

**Results:**

Pre-treatment with anti-TLR2 or anti-TLR4 mAb alone showed significant protection from sepsis-associated death. Protective effects were observed even when the administration of either anti-TLR2 or anti-TLR4 alone was delayed (i.e., 3 h after sepsis induction). Delayed administration of either mAb in combination with antibiotics resulted in additive protection.

**Conclusion:**

Although attempts to translate preclinical findings to clinical sepsis have failed so far, our preclinical experiments strongly suggest that there is a sufficient therapeutic window within which patients with ongoing sepsis could benefit from combined antibiotic plus anti-TLR2 or anti-TLR4 mAb treatment.

## Introduction

Sepsis, including severe sepsis and septic shock, is the end result of complex interactions between infecting organisms and host responses that is associated with high mortality rates in intensive care units. Although some novel therapeutic agents have recently been introduced in the field of sepsis [1–3], their clinical efficacy remain controversial [1, 4], as the mortality rate remains unacceptably high and they are associated with significant side effects [5–7]. Hence, further investigation into the mechanisms involved in the transition from infection to multiple organ damage and failure are required if effective future therapies are to be developed.

Toll-like receptors (TLRs) play an important role in the recognition of exogenous and endogenous danger signals [6]. TLR engagement gives rise to rapid activation of signaling pathways, such as those involving MAPK, NF-**κβ** and/or IFN responsive factors [8], thereby leading to the secretion of pro-inflammatory cytokines, reactive oxygen species, antimicrobial peptides and acute-phase proteins. Although signaling through TLRs is an important element of host defense, a growing body of evidence indicates that dysregulation of these receptors may also play a role in the pathogenesis of sepsis [7, 9, 10]. The massive release of inflammatory mediators into the bloodstream following TLR activation is suspected to be associated with sepsis, culminating in multiple organ failure.

Studies using genetically mutant or modified mice have demonstrated the detrimental effect of TLR signaling in sepsis-associated inflammatory responses [11–13]. Our group and others have shown that blockade of TLR2 or TLR4 was successful in decreasing disease severity in sepsis models of Gram-negative and-positive bacteria, respectively [10, 14, 15]. An anti-TLR4/MD2 monoclonal antibody (mAb) was also shown to decrease lethality in a model of polymicrobial sepsis caused by implantation of a stent in the cecum of mice (15). In this latter study, mechanisms of protection from death after polymicrobial sepsis were not investigated. However, these findings have raised the possibility that pharmacological modulation of TLR activation or downstream signaling pathways could become a useful approach in the control of overwhelming inflammatory responses of infectious origin after specific or polymicrobial bacterial infections. Indeed, it has been argued that certain aspects of the inflammatory response against infection may be amenable to therapeutic intervention without altering the ability of the host to deal with infection [16]. In the present study, we have investigated the effects of preventive and therapeutic administration of anti-TLR2 and anti-TLR4 mAbs, either alone or in combination, and in conjunction with antibiotic treatment in a model of polymicrobial sepsis caused by cecal ligation and puncture (CLP). Induction of sepsis by CLP is a standard and accepted model of polymicrobial sepsis that mimics several parameters observed in septic patients [17, 18]. Although pre clinical studies have failed to translate biological findings into effective clinical therapies, pre-clinical experiments may suggest novel therapies to be evaluated in patients and try to provide explanations for failures of clinical trials, such as the phase 3 clinical trial with eritoran tetrasodium, a MD2-TLR4 antagonist [19]. Our results demonstrate that blockade of either TLR2 or TLR4 significantly decrease systemic inflammatory responses and lethality especially when used in combination with antibiotics, representing a specific preclinical setting at which blockers of TLR may be useful in patients with sepsis.

## Materials and Methods

### Mice

Eight- to twelve-week-old male C57BL/6 background mice were housed under standard conditions in a temperature-controlled room (23 ± 1°C) on an automatic 12-h light/dark cycle and had free access to commercial chow and water. The Local Ethics Committee on Animal Experimentation, (Comitê de Ética em Experimentação Animal–Federal University of Minas Gerais), approved all procedures described in this study.

### Sepsis induction

Mice (6–10 animals/group–specific groups are given in the respective experiments described in the Figures) were anesthetized with 100 mg/kg ketamine and 5 mg/kg xylazine and randomized to CLP or laparotomy (Sham) surgery. The CLP procedure involved a laparotomy and ligation of the cecum, distal to the ileocecal valve. The cecum was punctured twice with a 16 gauge needle to induce severe sepsis. Following the puncture a small amount of fecal matter was forcedly extruded from each puncture. Sham animals received a laparotomy with manipulation of the cecum. Following ligation and puncture, the cecum was returned to the abdomen, the peritoneal wall and skin incisions were closed and the animals were allowed to recover. Immediately following CLP and at twelve hours post-op, animals were administered 1 ml of NaCl 0.9% subcutaneously. The survival rate was determined twice daily for 7 days after CLP induction. Animals were considered dead when weight loss was greater than 20% or when animals presented signs of severe morbidity. Death in this model of sepsis appears to be secondary to cardiovascular collapse, and multiple organ dysfunction [20]. In some experiments, animals were culled at determined time points (6 or 24 h after sepsis induction). To this end, animals were given twice the dose of anesthetic (ketamine/xylasine) and the relevant procedures performed. After all procedures, mice were submitted to neck dislocation to guarantee unnecessary suffering.

### Antibody treatment

In studies evaluating TLR2 or TLR4 blockade, 1 mg of each monoclonal antibody, T2.5 (anti-mouse TLR2; HyCult Biotechnology, Uden, Neitherlands) and 1A6 (anti-mouse TLR4; Novimmune SA) or their isotype-matched control (NovImmune SA, a mouse IgG1 anti-cMyc, clone number 9E10) were administered via subcutaneous injection either 45 min before (preventive schedule) or 3 h (therapeutic schedule) after sepsis induction. Equivalent volumes (200μL) of sterile 0.9% NaCl were used for antibodies administration. Doses of antibody used were based on previous published studies demonstrating these were sufficient to block the relevant receptors completely [10, 14].

### Antibiotic therapy

To mimic more closely the clinical situation in which there is a delay between insult and the onset of therapy, antibiotic therapy alone or paired with TLR blockade was initiated 3 h after CLP. A clinically relevant antibiotic regimen consisting of 12.5 mg/kg metronidazole and 25 mg/kg ceftriaxone was used, providing both aerobic and anaerobic coverage. Sham animals received equivalent volumes of 0.9% saline solution. All injections were administered intraperitoneally and were followed by gentle abdominal massage to insure complete mixing and distribution. Injections were repeated every 12 h and were continued for 7 days or until death.

### Bacterial Analysis

Bacterial counts were assessed as previously described [20]. Briefly, following sham or CLP surgery, animals were killed at 6 hours and blood was collected under sterile conditions and 10 μL of blood was plated on Muller-Hinton agar dishes (Difco Laboratories, Detroit, USA) and incubated at 37°C. Colony forming units (CFU) were analyzed after 24 h, and the results were expressed as CFU per mL of blood. For peritoneal lavage fluid, the peritoneal cavity was washed with 1.5 ml sterile PBS with 1mM ETDA. Aliquots were then serially diluted and plated on Muller-Hinton agar dishes and incubated at 37°C. CFU were analyzed after 24 h and results were expressed as log of CFU per peritoneal cavity.

### Neutrophil migration to the peritoneal cavity

Neutrophil migration to the peritoneum was assessed 6 hours following CLP. At sacrifice, the peritoneal cavity was washed with 1.5 ml of PBS containing 1 mM EDTA. Total cell counts were performed and differential cell counts were carried out on cytocentrifuge slides and stained by the May-Grünwald-Giemsa (Rosenfeld) method. The results are expressed as the number of neutrophils per cavity.

### Cytokine and chemokine quantification

Cytokines and chemokines were measured in the peritoneal lavage, plasma and lung tissue according to the procedures supplied by the manufacturer (R&D Systems, Minneapolis, MN). Briefly, 100 mg lung tissue was homogenized in 1 ml PBS (0.4 mol/l NaCl and 10 mmol/l NaPO_4_) containing antiproteases (0.1 mmol/l phenylmethyl sulfonyl fluoride, 0.1 mmol/l benzethonium chloride, 10 mmol/l EDTA, and 20 KI aprotinin A) and 0.05% Tween 20. The samples from lung homogenates, blood and peritoneal lavage were centrifuged for 10 min at 3000 × *g*, and the supernatants immediately used for ELISA assays at a 1:10 dilution in PBS.

### Statistical Analysis

Data are expressed as mean ± SEM. Multiple group differences were compared using ANOVA followed by Student-Newman-Keuls *post hoc* analysis. Survival was tested using the log-rank test, and bacterial counts were analyzed using the Mann-Whitney U test. A *P* value <0.05 was considered significant. Statistical analyses were performed using GraphPad Prism 4.0 for Windows (GraphPad Software, San Diego, CA, USA).

## Results

### Blockade of either TLR2 or TLR4 improves survival during polymicrobial sepsis

**Fig 1:**
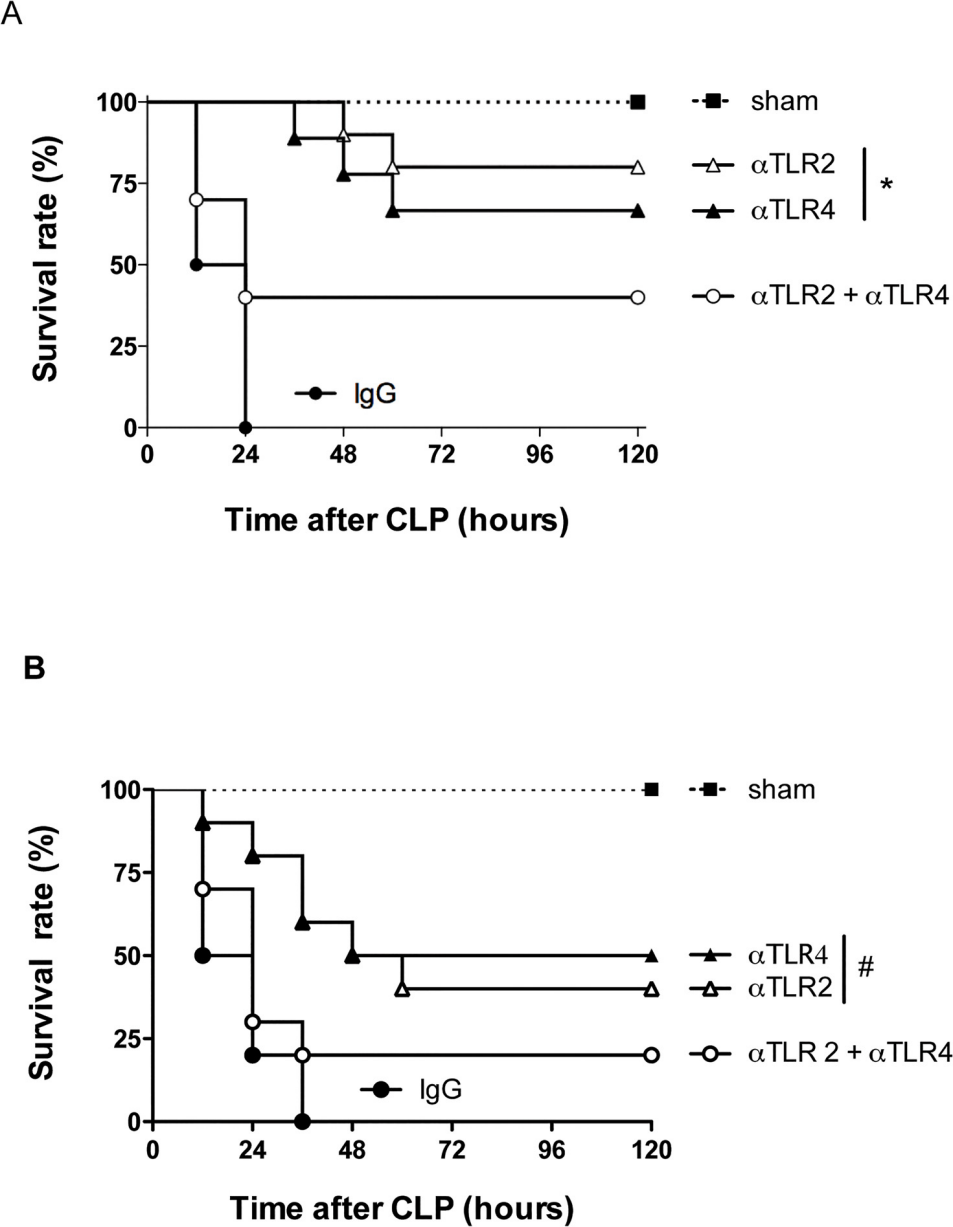
Blockade of either TLR2 or TLR4 improves survival during polymicrobial sepsis. The survival rate was analyzed following CLP procedure comparing the effect of the treatment with isolated or combined anti-TLR2 and anti-TLR4 antibodies. The treatments were performed 45 minutes before (A) or 3 hours after (B) the CLP procedure. The combined treatment with both anti-TLR2 and anti-TLR4 showed a similar survival curve compared to the negative control isotype IgG treated-group (CLP) * P<0.001 between Individual antibody treatments and negative control (IgG). # p<0.05 between individual antibody treatments and negative control (IgG) Antibody dose (1 mg/animal). (n = 10/group).

Preventive treatment with mAbs targeting either TLR2 (T2.5) or TLR4 (1A6) caused a significant improvement in the survival rate during severe sepsis (Fig 1A) compared to the isotype control group, where 100% of the mice subjected to CLP succumbed within 24 hours. Mice pre-treated (45 minutes before CLP) with either antibody had significantly enhanced survival rates, with 75 and 65% survival after treatment with anti-TLR2 or anti-TLR4, respectively (Fig 1A). Therapeutic administration of either anti-TLR2 or anti-TLR4, i.e., 3 hours after the CLP procedure, also provided significant protective effects with delayed lethality and approximately 50% protection (Fig 1B). Of note, in this particular experiment, approximately 50% of control antibody-treated mice succumbed 12 h after CLP. Paradoxically, when both antibodies were administered simultaneously to mice subjected to CLP, either prophylactically or therapeutically, there was only partial protection which was significantly lower than that afforded by either antibody given alone (Fig 1). A series of experiments were then conducted to evaluate the effects of individual or combined pretreatment with antibodies on inflammatory and infection indices induced by CLP.

### Blockade of either TLR2 or TLR4 improves inflammatory and infection indices in polymicrobial sepsis

**Fig 2:**
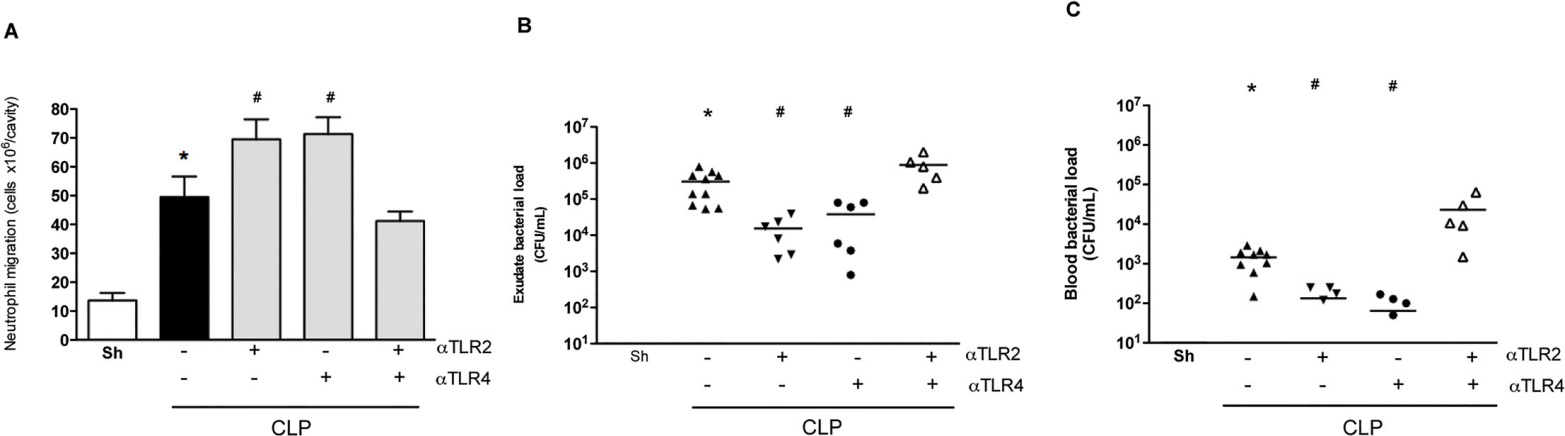
Blockade of either TLR2 or TLR4 improves inflammatory and infection indices in polymicrobial sepsis. Analysis of parameters of mice pre-treated (45 minutes before the CLP procedure) with individual anti-TLR2 or anti-TLR4 antibodies showed an increased neutrophil migration to the peritoneal cavity (A) and a decreased number of bacteria in peritoneum (B) and serum (C) 6 hours after sepsis compared to negative isotype IgG antibody treatment (black bar). The combined treatment with both anti-TLR2 and anti-TLR4 showed a similar inflammatory response compared to the negative control isotype IgG treated-group (CLP). * p<0.05 between the CLP and Sham groups. # p<0.05 between individual antibody treatments and negative control isotype IgG treated-group. Antibody dose (1 mg/animal). Horizontal bars in Fig 2A/2B represent median value. Used 6 animals/group, except isotype IgG antibody treated group (n = 10). Missing points in Figs reflect number of bacteria below detection limit.

Neutrophils play a crucial role in bacterial sepsis, as they have a series of effector functions that are relevant to bacterial clearance [20]. A previous study from our group has demonstrated that the failure of neutrophils to migrate early in response to an infectious challenge contributes substantially to the pathogenesis of polymicrobial sepsis induced by CLP [12]. Strategies which ameliorate neutrophil influx following CLP commonly induce better control of infection resulting in diminished lethality rates [20–22]. Pre-treatment of mice with either anti-TLR2 or anti-TLR4 resulted in an increase in the influx of neutrophils to the peritoneal cavity of mice subjected to severe CLP (Fig 2A). The increased neutrophil influx was associated with decreased numbers of bacteria in the peritoneal cavity (Fig 2B) and decreased spread of bacteria to the blood (Fig 2C). Co-treatment of mice with both mAbs prior to induction of CLP neither induced a significant increase in neutrophil accumulation nor altered bacterial growth in the peritoneal cavity, when compared to the control group (Fig 2A and 2B). Interestingly, there were significantly more bacteria in the blood of animals treated with the combination of anti-TLR2 and anti-TLR4 mAb than control antibody-treated animals (Fig 2C).

**Fig 3:**
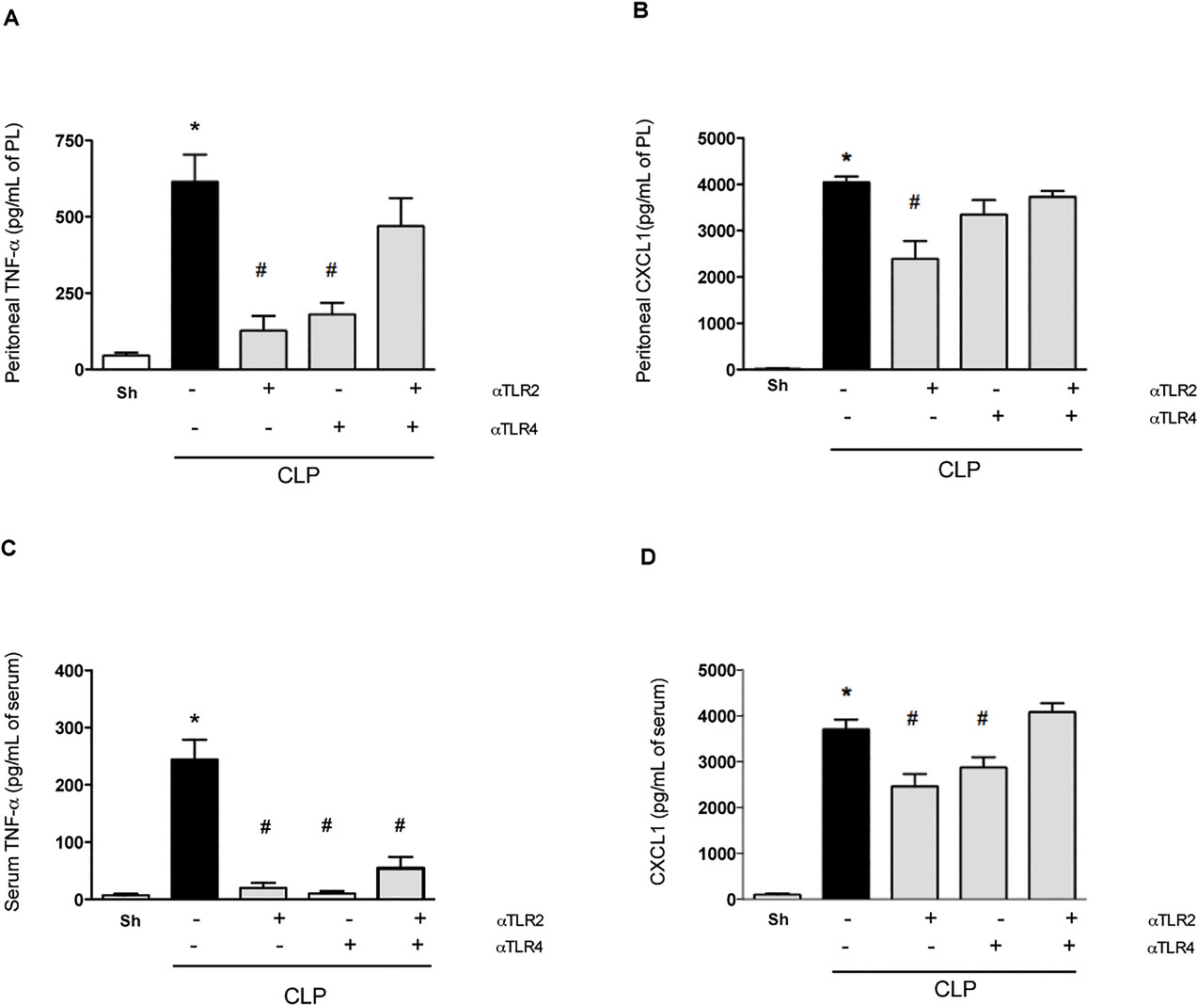
Blockade of either TLR2 or TLR4 reduces TNF-α and CXCL1 levels after polymicrobial sepsis. Mice were pre-treated with individual or combined anti-TLR2 or anti-TLR4 antibodies (45 minutes before the CLP procedure). Single treatment resulted in lower levels of TNF-α and CXCL1 (A and B, respectively) in the peritoneal cavity (PL: peritoneum lavage) and in serum (C and D, respectively) 6 hours after sepsis compared to the negative control isotype IgG antibody treatment (black bar). The combined treatment with both anti-TLR2 and anti-TLR4 presented similar response compared to negative isotype IgG treated-group. * p<0.05 between the CLP and Sham groups. # p<0.05 between the antibody treatment groups and negative control isotype IgG treated-group. Antibody dose (1 mg/animal). Used 6 animals/group, except isotype IgG antibody treated group (n = 10).

Induction of CLP was accompanied by significant increases in the levels of TNF-**α**, IL-1**β** and the neutrophil-active chemokines CXCL1 (Fig 3A and 3B) and CXCL2 (data not shown) in the peritoneum at 6 h post surgery. Also, an increase in systemic levels of TNF-**α** and CXCL1 (Fig 3C and 3D) was observed. Pre-treatment with either anti-TLR2 or anti-TLR4 mAbs alone prevented the increase of these cytokines and chemokines (Fig 3). In contrast, combined pre-treatment with both mAbs tended to have little effect on the levels of inflammatory mediators, with the exception of a significant fall in the levels of TNF-**α** in serum (Fig 3), which may account for the small decrease of lethality observed (Fig 1).

**Fig 4:**
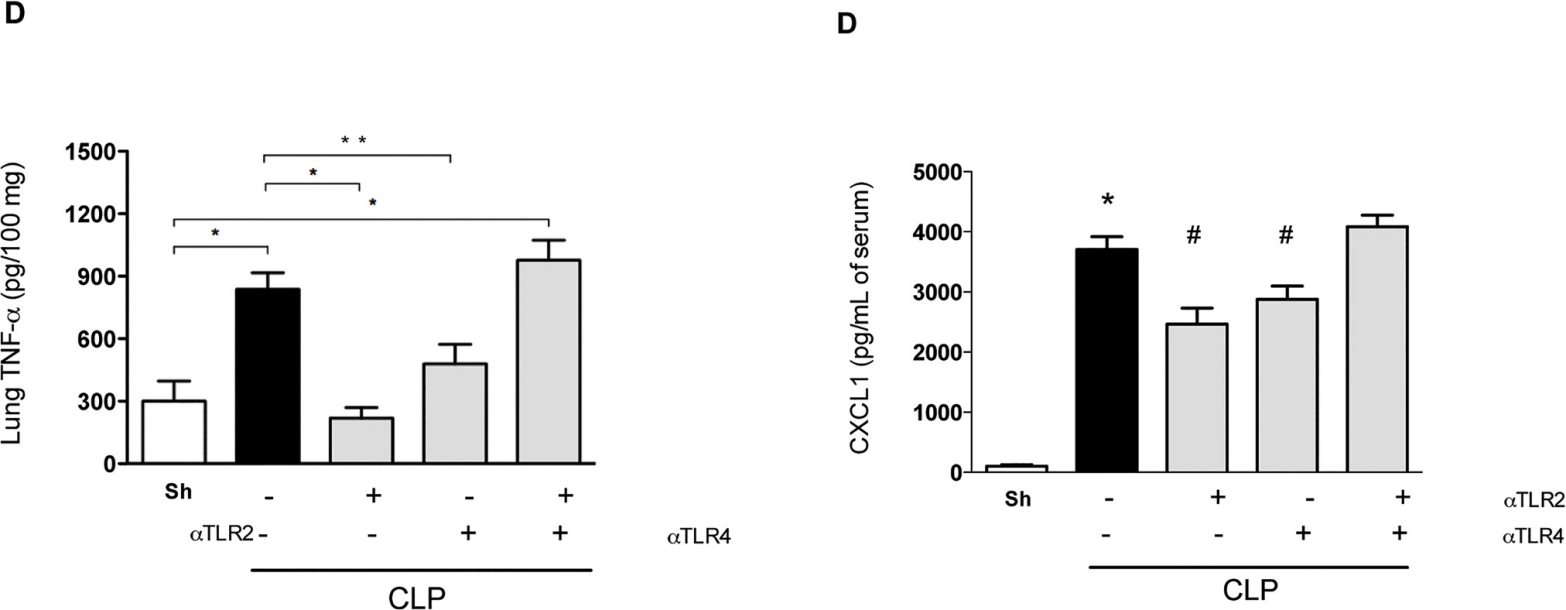
Blockade of either TLR2 or TLR4 reduces lung inflammation after polymicrobial sepsis. Mice were pre-treated with individual or combined anti-TLR2 or anti-TLR4 antibodies (45 minutes before the CLP procedure). Single treatment resulted in lower levels of TNF-α (A), IL-1β (B), CXCL1 (C) and myeloperoxidase in the lung 6 hours after sepsis compared to the negative control isotype IgG antibody treatment (black bar). The combined treatment with both anti-TLR2 and anti-TLR4 showed moderate improvement when compared to the control treatment-group. * p<0.05 between the CLP and Sham groups. # p<0.05 between the antibody treatment groups and negative control isotype IgG treated-group. Antibody dose (1 mg/animal). Used 6 animals/group, except isotype IgG antibody treated group (n = 10).

Inflammatory parameters in the lung, the target organ most commonly affected by systemic inflammatory responses [23], were also evaluated. Pre-treatment with either anti-TLR2 or anti-TLR4 mAbs diminished inflammation in the lung, as seen by the local production of IL-1**β**, TNF-**α**, and CXCL1, as well as the local accumulation of neutrophils (Fig 4). Again, and in contrast to the effects of individual treatment, combined pre-treatment with anti-TLR2 and anti-TLR4 mAbs failed to ameliorate pulmonary inflammation (Fig 4).

**Fig 5:**
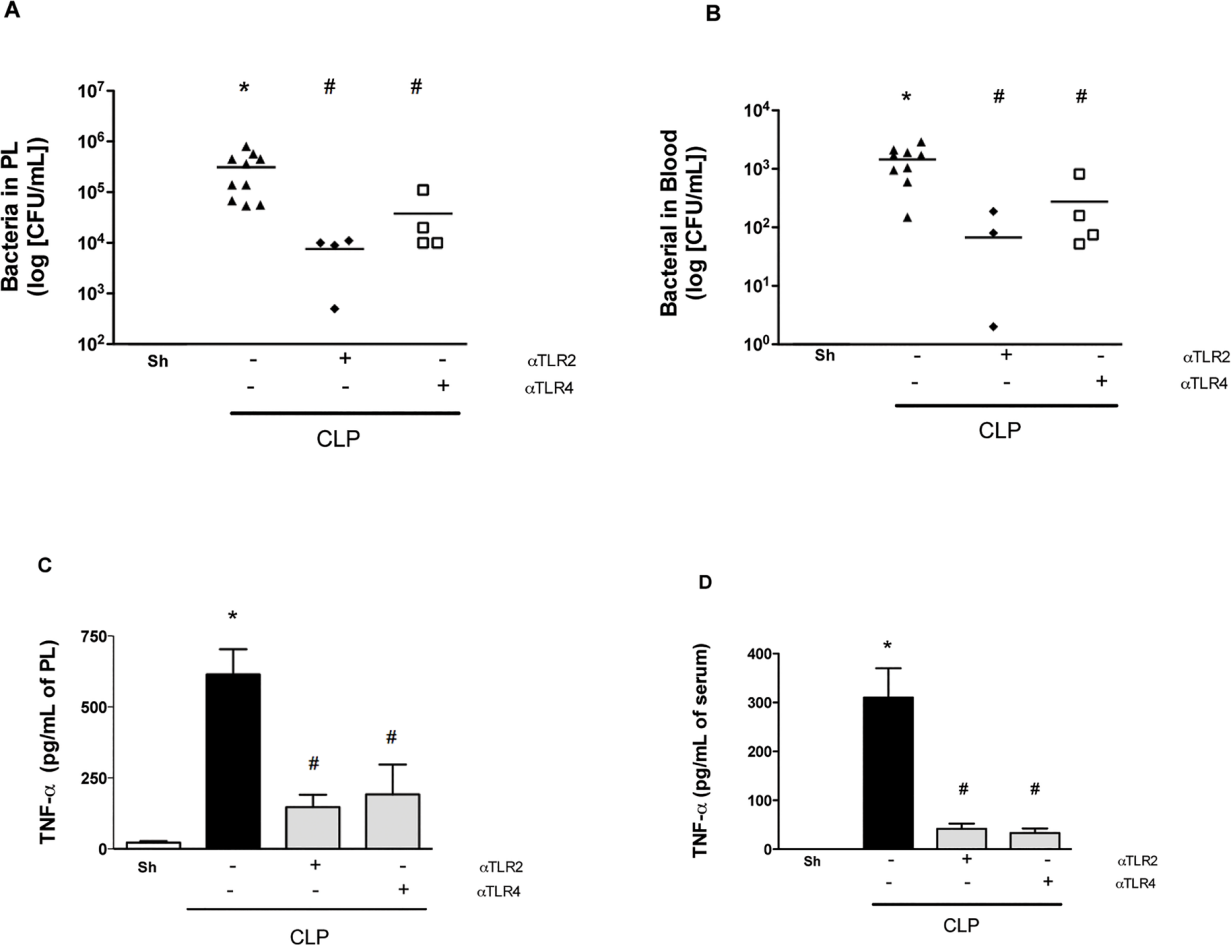
Therapeutic treatment with anti-TLR2 and anti-TLR4 improve sepsis. Mice were treated with individual anti-TLR2 or anti-TLR4 antibodies 3 hours after the CLP procedure. Treatment with both antibodies in combination decreased the number of bacteria in the peritoneum (A) and serum (B) as well as the levels of TNF-α in the peritoneum (C) and serum (D) 6 hours after sepsis compared to the negative control isotype IgG antibody treatment (CLP). * p<0.05 between CLP and Sham groups. # p<0.05 between the antibody treatment and negative control isotype IgG treated-group. Horizontal bars in Fig 5A/5B represent median value. Antibody dose (1 mg/animal). Used 6 animals/group, except isotype IgG antibody treated group (n = 10). Missing points in Figs reflect number of bacteria below detection limit.

As shown in Fig 1, therapeutic treatment with either mAb 3 h after CLP induction decreased lethality rates by approximately 50%. In the next set of experiments, the bacterial load and inflammatory parameters were analyzed for these therapeutic regimens. Treatment with either anti-TLR-2 or anti-TLR4 mAbs significantly decreased bacterial growth and spread at 6 h (Fig 5A and 5B) which was associated with significantly lower levels of TNF-**α** in the peritoneum and serum (Fig 5C and 5D). Therefore, either preventive or therapeutic treatment with either anti-TLR2 or anti-TLR4 mAbs, but not in combination, diminished local and systemic inflammatory responses and protected against polymicrobial sepsis.

### Combined effects of anti-TLR2/TLR4 and antibiotics in polymicrobial sepsis

**Fig 6:**
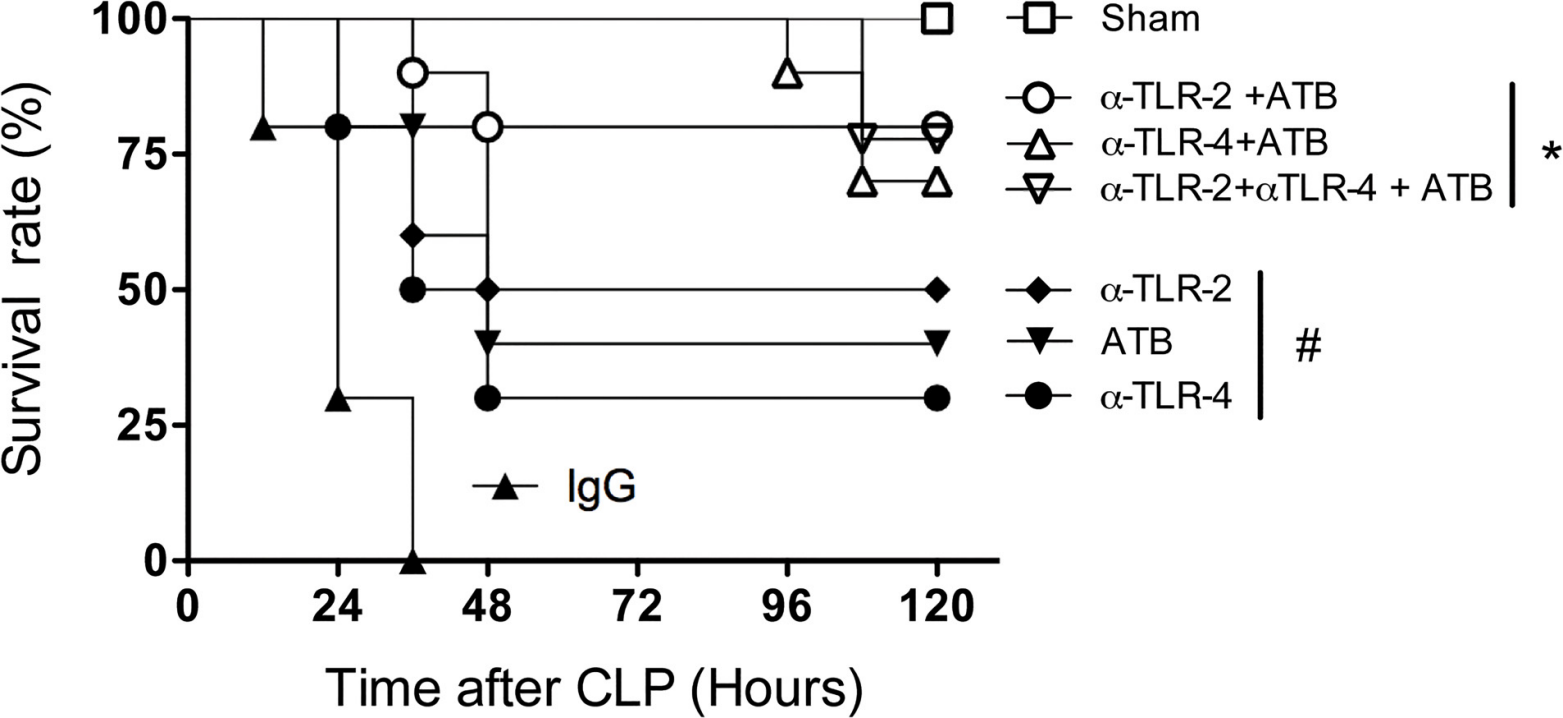
Combined effects of anti-TLR2, anti-TLR4 and antibiotics in polymicrobial sepsis. Mice were therapeutically treated with anti-TLR2, anti-TLR4 and antibiotics (metronidazole and ceftriaxone) separately or concomitantly 3 hours after the CLP procedure. The negative control isotype IgG antibody treatment was used as a control. n = 9–10 in each experimental group. * p<0.001 between use of the antibodies plus antibiotics and isotype IgG control and p<0.05 between individual use of antibodies or antibiotics. # p<0.05 between individual antibodies or antibiotics use and isotype IgG control. There was no difference in survival rates between mice given antibodies and antibiotics and the sham group (n = 10 animals/group). Antibody doses (1 mg/animal).

In the clinical situation, aggressive antibiotic therapy directed to the potential site of infection and initiated as soon as possible following diagnosis is recommended to treat septic patients [24]. In order to model this, antibiotics (metronidazole and ceftriaxone) were administered at different times (1, 3, 6, 12 and 24 h) post-induction of sepsis either alone or in combination with the various mAbs. A substantial protection was evident if treatments were administered until 3h after sepsis induction. Treatment after 3 h of sepsis initiation did not protect animals significantly (data not shown). As shown in Fig 6, antibiotic treatment alone decreased lethality rates to approximately 40%. These results were similar to those obtained with anti-TLR2 or anti-TLR4 mAbs alone in the absence of antibiotics (see Fig 1). The combination of antibiotics with anti-TLR2 or anti-TLR4 mAbs resulted in greater protection with approximately 75% of animals surviving at day-5 post-CLP induction (Fig 6). Interestingly, the combination of anti-TLR2 and anti-TLR4 mAbs in the presence of antibiotics also resulted in significant protection (75%) (Fig 6), which was in stark contrast to that observed in the absence of antibiotics (Fig 1). Overall, it is clear that administration of antibiotics with one of the antibodies (anti-TLR2 or anti-TLR4) provides better than or similar protection to administration antibiotics with both antibodies together. Delayed treatment also affected neutrophil recruitment; suggesting reversal of neutrophil paralysis is relevant for the mechanism of action of these molecules (data not shown).

## Discussion

TLR2 and TLR4 are components of the innate immune response and play a crucial role in host defense against Gram-positive and Gram-negative bacterial infection. Despite the protective role of innate immune responses, massive activation of inflammatory pathways and release of inflammatory mediators into the bloodstream are clearly detrimental in severe sepsis. Previous studies by our group showed that either TLR2- or TLR4-deficient mice display improved bacterial clearance and reduced mortality during polymicrobial sepsis [12, 25]. It has also been shown that systemic administration of T2.5 upon lipopeptide challenge inhibited release of inflammatory mediators such as TNF-**α** and prevented lethal shock-like syndrome in mice [10]. In addition, the blockade of TLR2 and TLR4 with the mAbs T2.5 and 1A6, respectively, prevented otherwise fatal shock in experimental *Salmonella enterica* or *Escherichia coli* infection models [14]. However, this is the first study to compare the effects of single or combined administration of mAbs that block TLR2 or TLR4 with or without antibiotics in polymicrobial sepsis. Moreover, the present study evaluates the therapeutic potential of these strategies when given after onset of disease.

We demonstrate a clear protective effect of the treatment with mAbs against TLR2 or TLR4 in a model of severe polymicrobial septic peritonitis induced by CLP. Our major findings can be summarized as follows: (i) the blockade of a single receptor (i.e., TLR2 **or** TLR4) enhanced local neutrophil influx, decreased bacterial load, diminished systemic and pulmonary inflammatory responses and decreased lethality; (ii) protective effects were observed even when the administration of mAbs was delayed (ie. 3 h after sepsis induction but not later); (iii) delayed administration of either mAb in combination with antibiotics resulted in additive protection with approximately 75% survival; (iv) combined administration of both anti-TLR2 and anti-TLR4 resulted in little protection when compared to animals given control mAbs. Indeed, combined administration of mAbs was less effective than administration of either mAb alone, resulting in a detrimental situation for the animal. Indeed, when the mAbs were combined, while local neutrophil influx was still decreased, both systemic inflammation (with the exception of lower levels of TNF-**α**) and bacterial spread were maintained, resulting in high lethality rates.

Our group has previously shown that the failure of neutrophils to migrate to the site of infection is associated with a worse outcome following severe sepsis [26]. Indeed, strategies that reconstitute neutrophil migration are associated with better control of local infection, prevention of bacterial spread and better outcome/survival [22]. Mechanistically, in severe sepsis, components of bacteria (such as TLR2 and TLR4 ligands) or mediators of inflammation may reach the circulation and activate circulating neutrophils leading to loss of CXCR2 from the surface of neutrophils [26]. As CXCR2 is crucial for neutrophil influx in response to polymicrobial infection, decreased expression of this receptor is associated with decreased neutrophil influx, increased bacterial growth and greater lethality. Therefore, in severe sepsis, it is not the lack of chemoattractants that explain the failure of neutrophils to migrate. Indeed, levels of chemoattractants are enhanced as we observed in our studies. It is the loss of CXCR2 that associates with the failure of neutrophils to migrate [26]. TLR2 or TLR4-deficient mice are more resistant to polymicrobial sepsis. In these animals, CXCR2 is not lost from the surface of neutrophils and recruitment of this cells is sufficient to control infection,–i.e., there is no failure of neutrophils to migrate [12, 27]. In our experiments, pre-treatment with either anti-TLR2 or anti-TLR4 alone restored neutrophil migration, decreased local bacterial growth and prevented bacterial spread, resulting in decreased systemic inflammation and lethality. These results are similar to those previously reported using TLR2- and TLR4-deficient mice and suggest that restoration of neutrophil migration by treatment with mAbs is the mechanism by which these reagents protect mice from polymicrobial sepsis. It is likely that during an infection in which multiple TLR-stimulating bacterial ligands are present, the absence of signalling via one TLR is insufficient to impair the induction of a local inflammatory response, but sufficient to prevent systemic inflammation and neutrophil “immune paralysis”.

Previous study of Roger and cols. (26) described that single treatment with anti-TLR4 is protective when administrated up to 13 hours after E.coli sepsis in conjunction with antibiotics. Our results demonstrated that the single use of mAb therapy reduced death from polymicrobial sepsis even when treatment was delayed for up to 3 h post-sepsis induction, but not at later time points. The difference in outcomes may be justified by the lower *E*. *coli* inoculum used in that study that caused an acute but less fulminant course of sepsis. Together, these two studies do suggest that there may be a therapeutic window for the treatment of septic patients with anti-TLR2 or anti-TLR4 mAbs. In severe polymicrobial sepsis caused by CLP, the therapeutic window was quite narrow.

Current clinical guidelines emphasize the importance of aggressive and standardized critical care support in sepsis treatment. The established therapeutic approach for patients with sepsis is based on early administration of appropriate antibiotics [24]. To mimic this situation, animals subjected to severe CLP were given antibiotics 3 h after induction of sepsis and this resulted in protection of approximately 40% of animals. Importantly, co-administration of antibiotics with either anti-TLR2 or anti-TLR4 resulted in relevant improvement of survival. Therefore, our preclinical experiments strongly suggest that there exists a therapeutic window in which to use anti-TLR2 or anti-TLR4 mAbs with antibiotics in patients with ongoing sepsis.

Contrary to TLR2- or TLR4-deficient mice, mice which are deficient in Myd88, a signalling module shared by many TLR receptors, failed to deal with mild or moderate sepsis and succumbed rapidly to infection [28]. In Myd88-deficient animals, local inflammatory responses are reduced, leading to significant spread of infection, overwhelming systemic inflammation and consequent death of the animals. In the current experiments, we have used a model of very severe sepsis with significant local bacterial growth, bacteremia and lethality rates. In our experiments, contrary to single mAb administration, concomitant blockade of both TLR2 and TLR4 was not associated with protection from severe sepsis. Indeed, there was a failure of neutrophils to migrate, coupled with significant local bacterial growth and exacerbated systemic spread of bacteria. This resulted in significant local, systemic and pulmonary inflammatory responses (with exception of decreased systemic TNF- **α**) and death of the animals. However, when animals treated with both anti-TLR2 and anti-TLR4 were given antibiotics, there was further decreased lethality compared to either single antibody therapy or antibiotic therapy alone. Therefore, it appears that activation of TLRs have two distinct role in sepsis. First, activation of either TLR2 or TLR4 is sufficient to drive efficient local inflammatory responses that will eventually prevent systemic inflammation and control severe polymicrobial infection, as observed in TLR-2 or TLR-4-deficient animals or those given a single mAb. Second, their combined action is, however, necessary to induce severe systemic inflammation, failure of neutrophils to migrate and to cause death of the animals. Indeed, blockade of one of the TLRs is sufficient to decrease this systemic response. In the triple treated animals (i.e., both mAbs plus antibiotics), bacterial spread is controlled by the antibiotics while the blockade of the TLR2 and TLR4 pathways are now relevant only to control systemic inflammation.

In contrast with with our studies, It there has been disappointing recent results from a phase 3 clinical trial with eritoran tetrasodium, a MD2-TLR4 antagonist [19] In this trial, treatment with eritoran was not beneficial in sepsis when compared to treatment controls. Those results differ significantly from our and other preclinical studies that show beneficial effect of TLR4 blocked or absence in the context of sepsis. It is not simple to reconcile these experiments in the preclinical setting and those in patients but several experimental differences can be pointed out. Experiments in mice are carried out with young mice (usually 8–12 weeks old) whereas most patients were > 65 in the eritoran trial. It’s well In the preclinical setting, a defined focus of sepsis is induced (in our experiments, abdominal polymicrobial sepsis) and effective antibiotics tailored to the model are given. Moreover, timing of treatment is carefully controlled from the onset of disease in the preclinical setting but from the diagnosis (associate with onset of symptoms) in the clinical setting. Our experiments clearly show that there is a therapeutic window that is narrow in severe sepsis. However, our results do suggest that early administration of anti-TLR2 or anti-TLR4 antibodies, especially with antibiotics, has a beneficial effects in the context of polymicrobial sepsis in the peritoneal cavity. Trying to extrapolate our findings to the human situation, it is possible that the use of the antibodies with antibiotics may be useful in certain subgroups of patients but is not likely to be useful in the general septic population. Further studies are necessary to ascertain which patients would benefit the most. In light of our findings, those with polymicrobial sepsis, younger and peritoneal cavity sepsis would benefit the most. In a recent published article, Cohen emphasized that the instead of undertaking all-comers trials of sepsis, investigators should study clinically and microbiologically defined groups with specific infections [29]. Indeed the application of real-time genomic (or proteomic) signatures might allow the identification of subsets of patients who are sufficiently homogeneous to allow intervention with specific drugs that are targeted to modify specific pathways of tissue injury. In a recent published review, Hotchkiss and Sherwood emphasized that sepsis is not only excessive inflammation, but there is also impaired immune responses to microbial infection and compensatory anti-inflammatory response syndrome that follows the excessive inflammation [30]. There is now much interest in understanding the two latter phenomena in sepsis as these may elicit novel therapies to be evaluated in humans.

## Conclusions

Despite a significant amount of fundamental research in the field of sepsis, there is clearly a need to advance our understanding in order to develop new therapies that improve patient outcomes. There have been no new therapies approved for septic shock treatment since recombinant human activated protein C in 2001 [3, 31] and even this therapy has been withdrawn, since it did not reduce mortality in septic shock [32]. The findings of the current study reinforce the role of TLRs in the pathogenesis of sepsis and suggest that blockade of TLR2 or TLR4 may be a useful therapeutic approach to control systemic inflammation, organ injury and lethality in polymicrobial sepsis. Importantly, when given in therapeutic schedule, the protective effects of antibodies on lethality were additive with antibiotic therapy (Fig 5). Our results confer a rationale for the blockade of TLR2 and/or TLR4, in addition to antibiotic therapy for the treatment of polymicrobial bacterial infection, and suggest this therapy may indeed be useful to specific subset of patients
